# Supplementary figures and images for: An ecological transcriptome approach to capture the molecular and physiological mechanisms of mass flowering in Shorea curtisii
Source: PeerJ. 2023 Nov 29;11:e16368. doi: 10.7717/peerj.16368 (PMC10693236; doi:10.7717/peerj.16368)

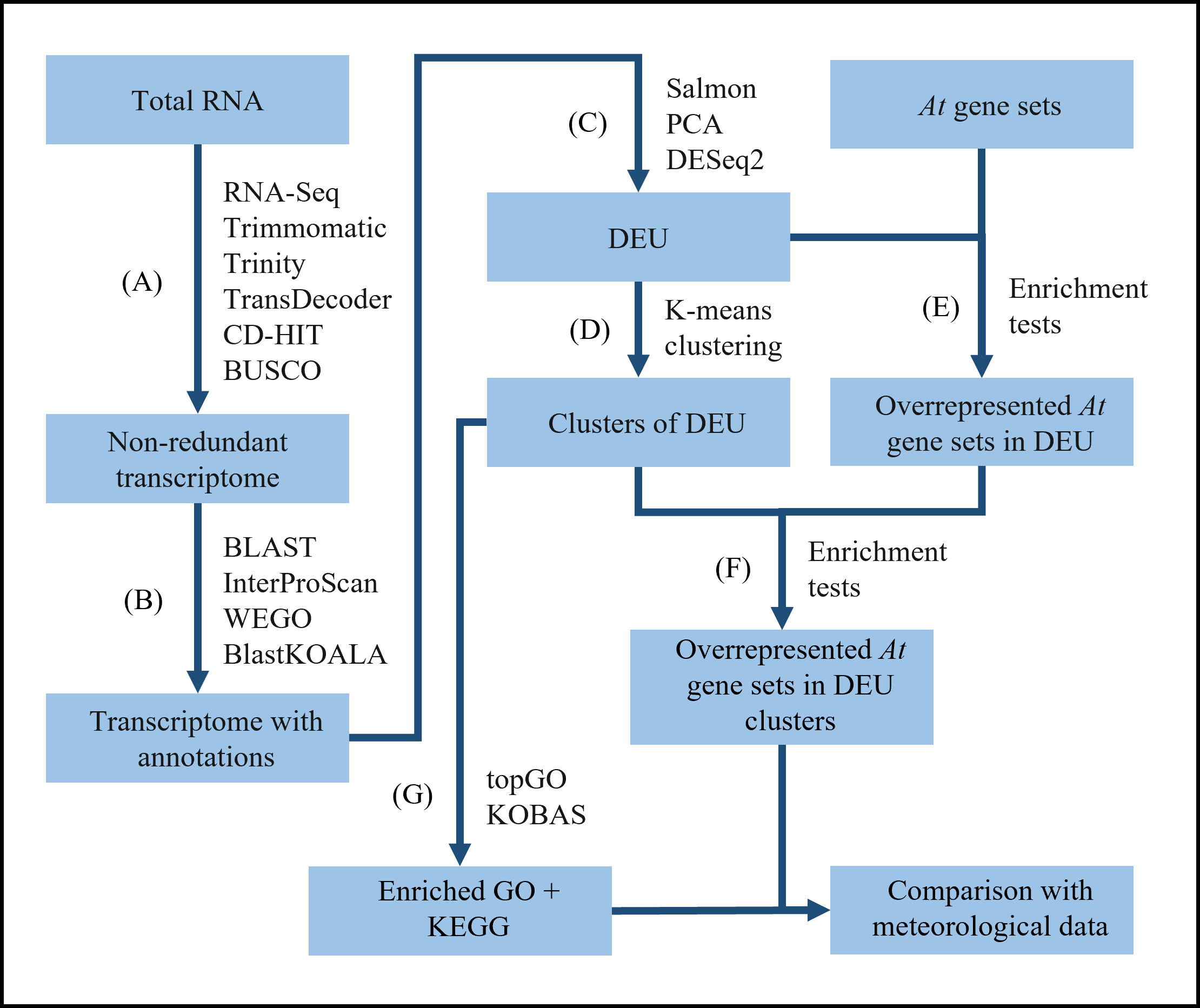

Supplement: Supplemental Information 1 — All the samples were pooled and analyzed together in steps A–C whereas the leaf and bud samples were analyzed separately for the rest of the bioinformatic pipeline (D–G). (A) Quality control and removal of low quality bases and adapters prior to de novo assembly. Coding regions within each transcript were predicted and only transcripts with complete open reading frame were retained. Highly similar sequences were clustered to reduce the number of transcripts. The completeness of the assembly was also assessed. (B) Annotation of the transcriptome using publicly available protein databases. (C) Quantification of transcripts followed by principal component analysis (PCA) and identification of differentially expressed unigenes (DEUs). (D) Expression profile analysis of the DEUs that were clustered based on their expression patterns. (E) Identification of significantly enriched A. thaliana gene sets in the DEUs using gene enrichment tests. (F) Identification of A. thaliana gene sets significantly enriched in specific DEU cluster. (G) Identification of significantly enriched GO terms and KEGG pathways in the DEUs. The enrichment analysis results (F and G) were then compared with the meteorological data, namely temperature records and cumulative rainfall data. Refer to the main text for a detailed description of the analyses. [file peerj-11-16368-s001.png]

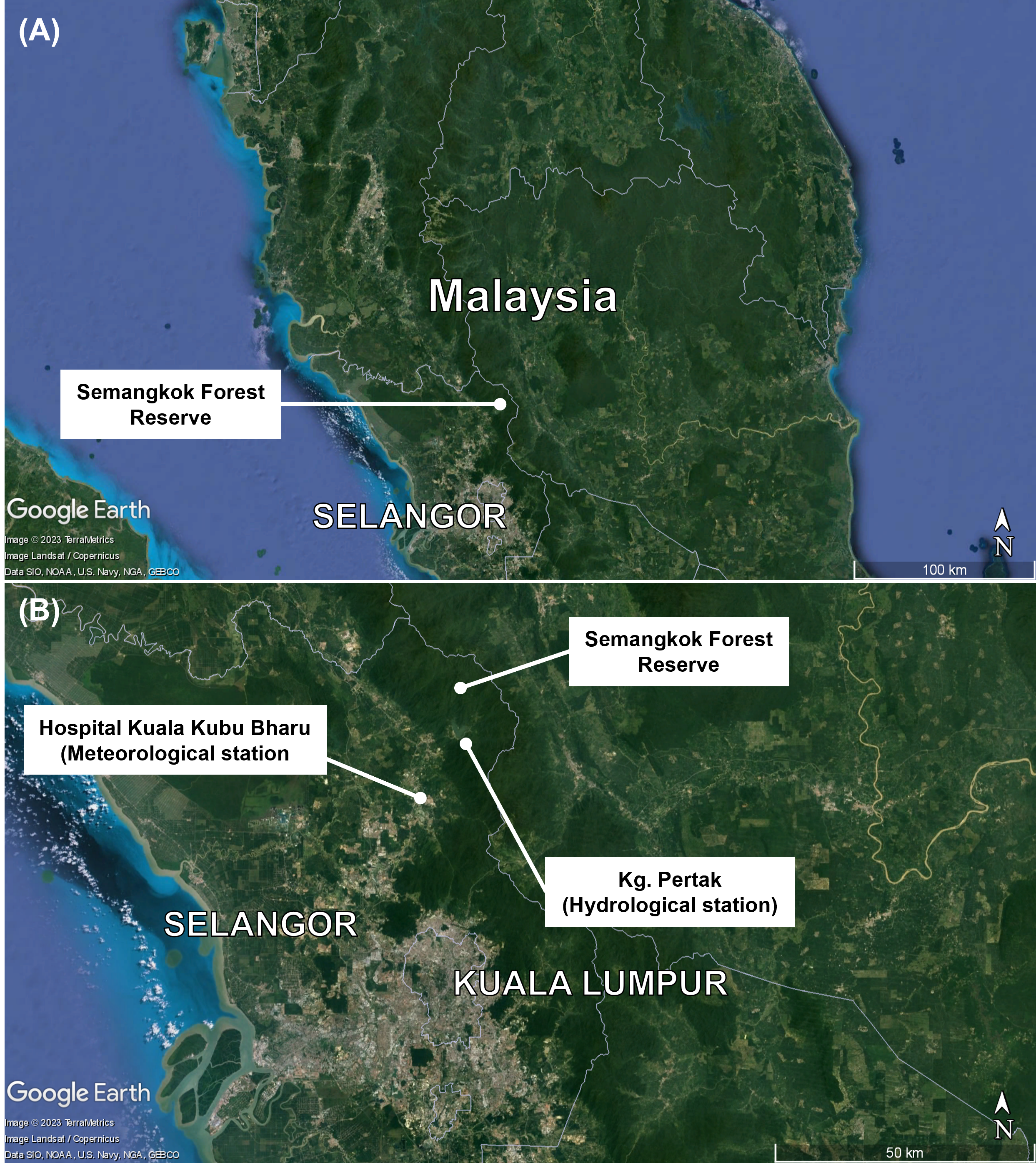

Supplement: Supplemental Information 2 — Image ©2023 TerraMetrics. Landsat/Copernicus. Map data: Data SIO, NOAA, US Navy, NGA, GEBCO [file peerj-11-16368-s002.png]

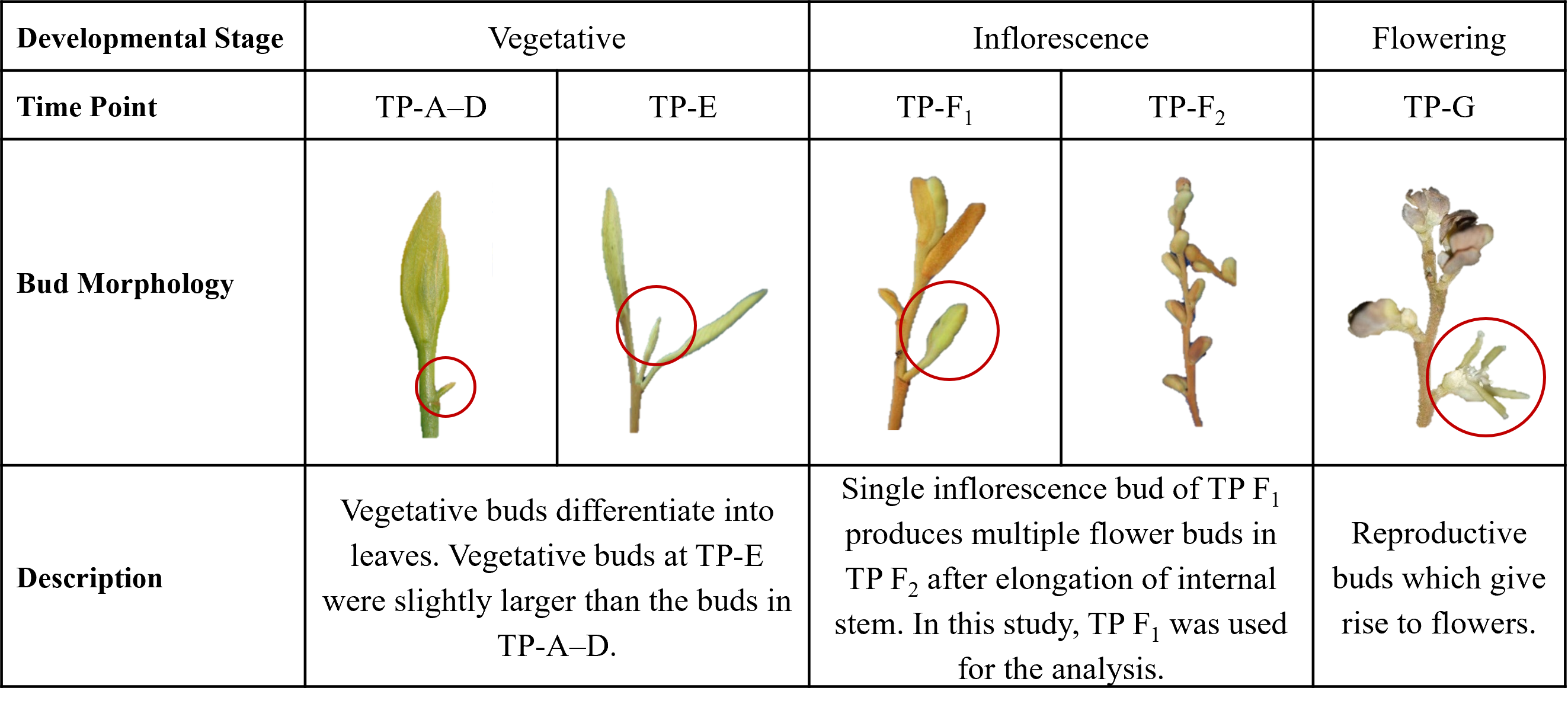

Supplement: Supplemental Information 3 — The developmental stages of the Shorea curtisii trees were determined based on the morphology of the buds collected at the corresponding time point (TP). [file peerj-11-16368-s003.png]

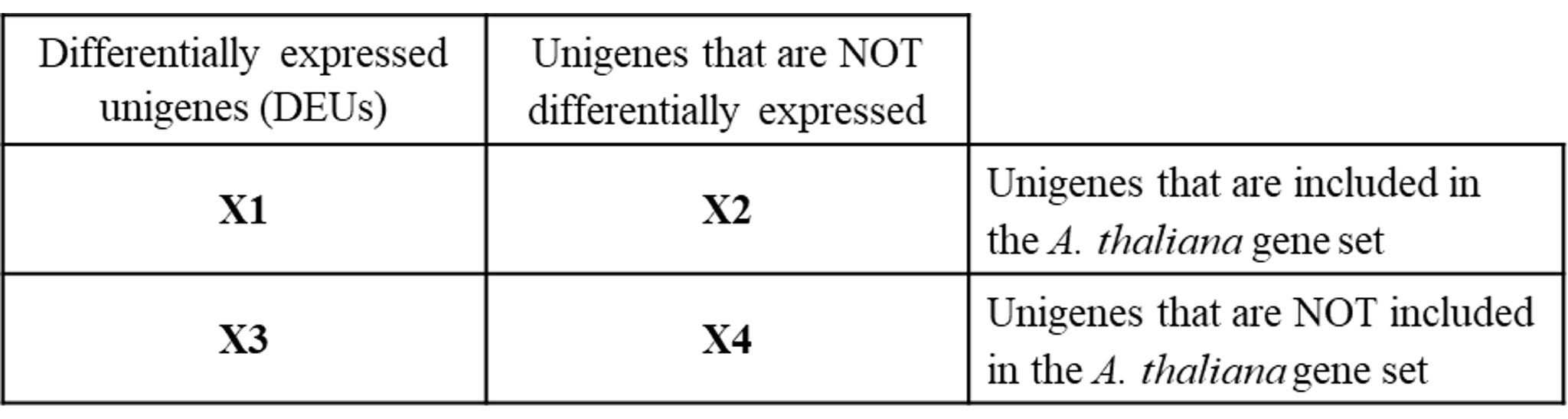

Supplement: Supplemental Information 4 — X1 = The number of DEUs that are in the A. thaliana gene set. X2 = (The number of unigenes that are included in the A. thaliana gene set) –X1. X3 = The number of DEUs that are not included in the A. thaliana gene set. X4 = (Total number of unigenes) –X1 –X2 –X3. [file peerj-11-16368-s004.png]

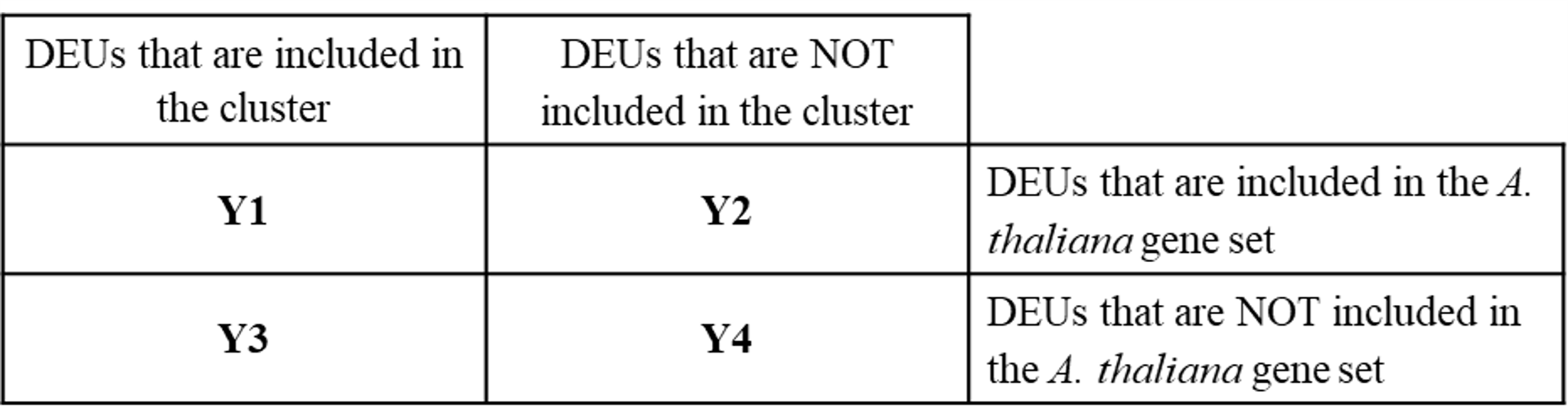

Supplement: Supplemental Information 5 — Y1 = The number of DEUs that are in the cluster and the A. thaliana gene set. Y2 = (The number of DEUs that are included in the A. thaliana gene set) –Y1. Y3 = The number of DEUs that are in the cluster but not in the A. thaliana gene set. Y4 = (Total number of DEUs) –Y1 –Y2 –Y3. [file peerj-11-16368-s005.png]

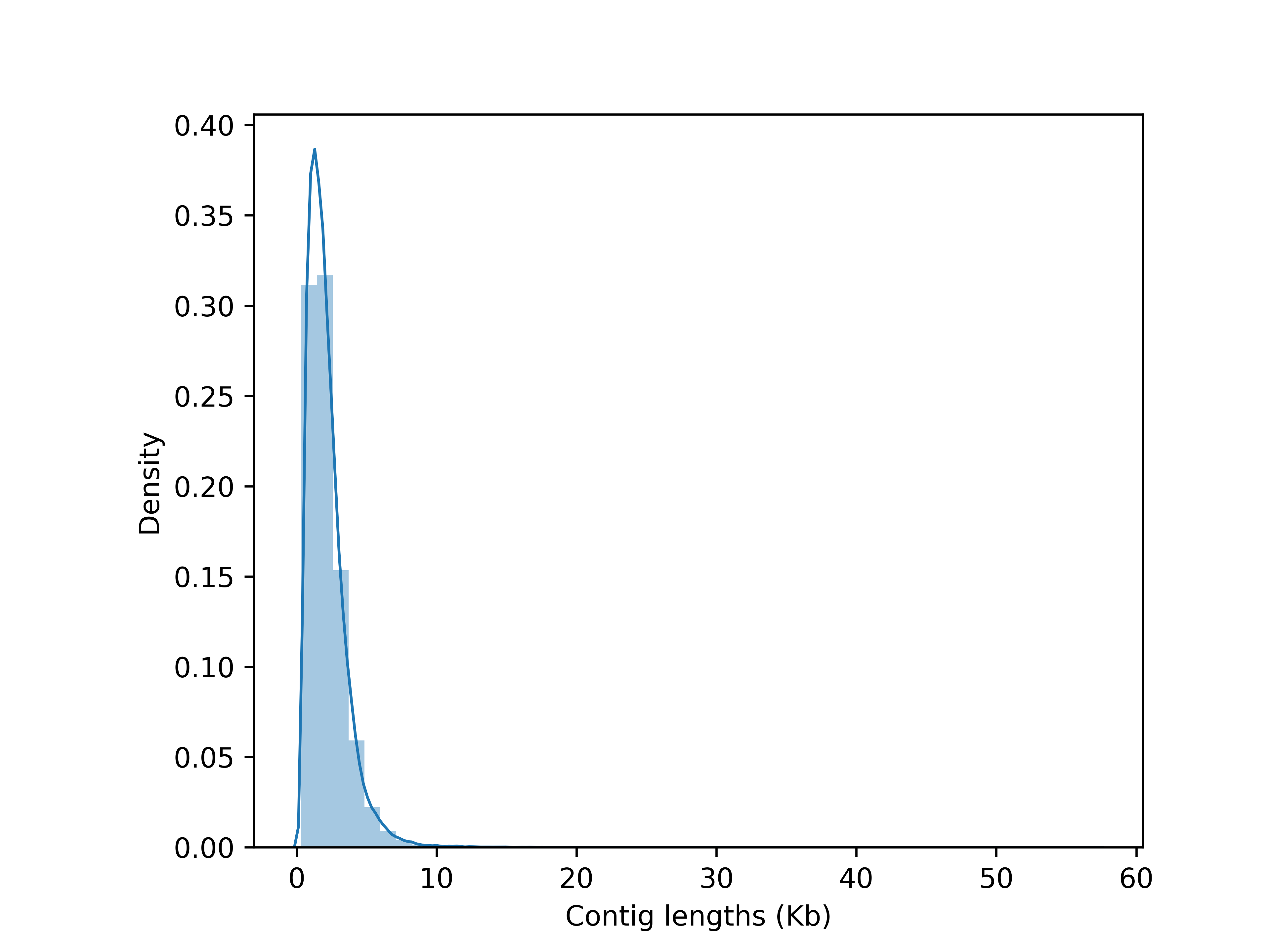

Supplement: Supplemental Information 6 [file peerj-11-16368-s006.png]

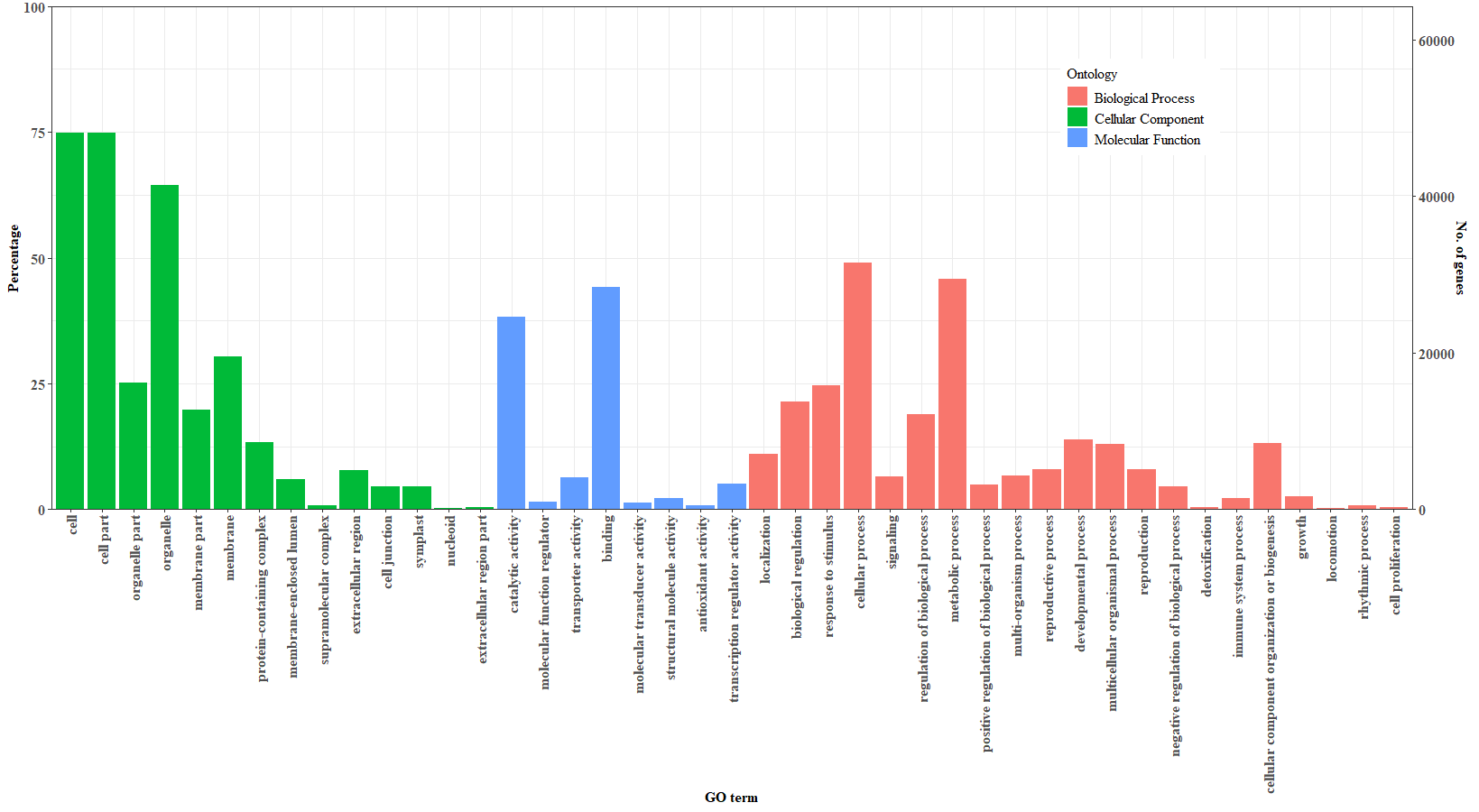

Supplement: Supplemental Information 7 — The x-axis shows the identified GO terms divided into three categories: cellular component, molecular function, and biological processes. The left y-axis indicates the percentage of unigenes (number of unigenes associated with the GO terms divided by the total number of unigenes) while the right y-axis shows the number of unigenes annotated with the GO terms. [file peerj-11-16368-s007.png]

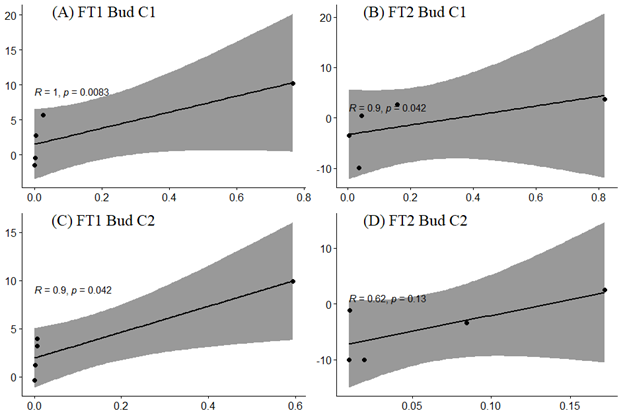

Supplement: Supplemental Information 8 — To validate the RNA-Seq results, the expression profile of key flowering gene homologues namely ScFT1 and ScFT2, were compared with the expression profile of the corresponding homologues obtained by qRT-PCR reported in an earlier study in S. curtisii (Yeoh et al., 2017). Scatter plots show simple linear regression and the correlation coefficient, R2 between qRT-PCR (x-axis) and log 2 of RNA-Seq read counts (y-axis). [file peerj-11-16368-s008.png]

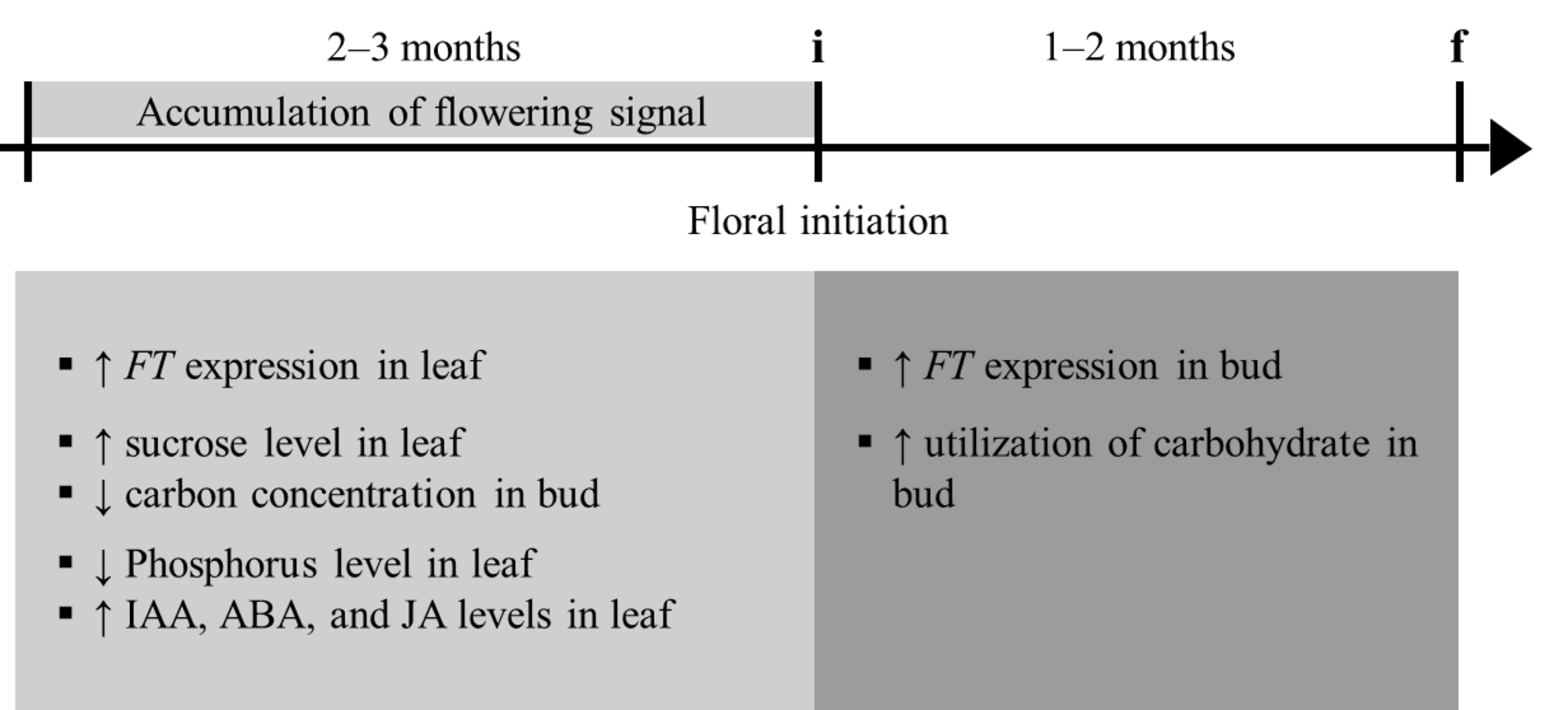

Supplement: Supplemental Information 9 — Accumulation of flowering signals, such as drought, begin approximately 2–3 months prior to floral initiation. In response to these stress signals, levels of phytohormones, including IAA, ABA, and JA are elevated. Concurrently, sucrose level in the leaf increases, while carbon concentration in the bud decreases, presumably in preparation for floral initiation. Expression of FT in the leaf gradually increases and peaks at floral initiation, followed by the emergence of inflorescence buds. Expression of FT in then the bud remains high until flowering time. Carbohydrate content in the bud decreases as the plant prepares for anthesis. I: Inflorescence/floral initiation; F: Flowering; ↑ : Upregulation, ↓ : Downregulation; IAA: Auxin; ABA: Abscisic acid; JA: Jasmonic acid; GA: Gibberellic acid. [file peerj-11-16368-s009.png]
